# Individual differences in dopamine-related traits influence mood effects of dopamine D2-antagonist and antidepressant treatment expectations

**DOI:** 10.1093/ijnp/pyaf067

**Published:** 2025-09-12

**Authors:** Li-Ching Chuang, Nick Augustat, Philipp Bierwirth, Ty Lees, Diego A Pizzagalli, Dominik Endres, Erik M Mueller

**Affiliations:** Department of Psychology, University of Marburg, Marburg, Germany; Department of Psychology, University of Marburg, Marburg, Germany; Department of Psychology, University of Marburg, Marburg, Germany; Center for Depression, Anxiety and Stress Research, McLean Hospital, Belmont, MA, United States; Department of Psychiatry, Harvard Medical School, Boston, MA, United States; Center for Depression, Anxiety and Stress Research, McLean Hospital, Belmont, MA, United States; Department of Psychiatry, Harvard Medical School, Boston, MA, United States; Noel Drury, M.D. Institute for Translational Depression Discoveries, University of California, Irvine, United States; Department of Psychology, University of Marburg, Marburg, Germany; Department of Psychology, University of Marburg, Marburg, Germany

**Keywords:** anhedonia, dopamine, depressive disorders, treatment expectations, positive affect

## Abstract

**Background:**

High trait anhedonia and low trait extraversion have both been previously related to not only low state positive affect but also depressive disorders, disrupted reward processing, and altered mesolimbic dopaminergic signaling. Research on placebo responses suggests that treatment expectations may alter dopamine signaling, elevate positive affect, and reduce depressive symptoms in anhedonic individuals. However, it remains unclear whether such antidepressant placebo responses depend on putative low baseline dopaminergic functioning in high anhedonia and low extraversion. The present study investigates how interindividual differences in these traits influence positive affective responses under manipulation of dopamine and treatment expectations.

**Methods:**

In a randomized, double-blind 2 × 2 design (*N* = 297), we administered either placebo or the dopamine D2 receptor antagonist sulpiride (400 mg), and manipulated treatment expectations by telling participants that they received either a mood-elevating drug or an inactive substance. Moreover, we assessed trait anhedonia and extraversion, and had participants rate their state positive affect at 6 different time points before and after treatment.

**Results:**

Trait anhedonia and extraversion, as well as a broad trait positive affectivity factor, predicted state positive affect across time points. Importantly, the effects of sulpiride and antidepressant treatment expectations on positive affect were moderated by dopaminergic traits such that sulpiride increased state positive affect in high anhedonia but decreased it in low anhedonia. Similarly, antidepressant treatment expectations raised positive affect in low extraversion but reduced it in high extraversion.

**Conclusions:**

This study demonstrates that dopamine-related individual differences moderate the effects of both sulpiride and a placebo intervention on positive affective state.

**Significance Statement**

In one of the first pharmacological studies examining the effects of treatment expectations and dopamine on mood in a large, healthy sample, we observed that lower baseline positive affectivity was linked to stronger mood-elevating treatment responses to both a placebo and a dopamine-related drug over time. This highlights how individual differences in relevant traits can influence treatment effectiveness, offering valuable insights for tailoring personalized approaches to depression care.

## INTRODUCTION

Human beings vary in their capacity to experience positive affect. Among healthy individuals, this variability is reflected across the expressiveness of traits like anhedonia,^1^^,^^2^ extraversion^3^^,^^4^ or broader dimensions of positive affectivity.^5^ Apart from being a trait with varying levels in the general population, low positive affect—or anhedonia—represents a cardinal symptom of depressive disorders, which rank among the most burdensome and disabling conditions globally.^6^^,^^7^

Individual differences in anhedonia,^8^ depression,^9^ and extraversion^10^^,^^11^ are presumably related to variations in dopaminergic functioning. Anhedonia reflects impaired motivation and reward processing, both of which are closely tied to dopamine.^1^^,^^2^^,^^8^^,^^12–14^ In line with this, substances that increase dopamine signaling are effective in treating depression.^15^^,^^16^ Similarly, several theories propose a close link between extraversion and dopamine-related brain functions,^10^^,^^17^ and some support for this assumption has emerged from pharmacological challenge studies linking questionnaire measures of extraversion to dopaminergic drug-evoked prolactin response.^18^

The individual experience of positive affect may further depend on one’s expectations. The influence of expectations on affect is powerfully demonstrated in antidepressant placebo responses, which have been reported in pharmacological and laboratory studies deliberately manipulating positive treatment expectations.^19^ Moreover, it has been assumed that positive treatment expectations involve endogenous dopamine^20^ and may be considered a type of reward response driven by expectations of clinical benefit. Some evidence of this emerges from Parkinson’s disease research, where placebo responses have been linked to dopamine release and the strength of treatment expectations.^21^ Further support stems from research linking reward system activation to placebo analgesia and its expectation.^22^^,^^23^ Interestingly, greater placebo-induced dopamine release has been observed in depression non-remitters.^24^

Notably, individual differences in anhedonia, extraversion, and the broader construct of positive affectivity have not only been conceptually linked to the dopaminergic system but have also been associated with the magnitude of placebo responses. These include optimism,^25^ extraversion,^26–28^ approach behavior,^29^ and personality traits related to dopaminergic neurotransmission.^30^ Moreover, individual variations in dopamine release in brain regions involved in reward encoding have been found to underlie placebo responses.^22^ Given the association between dopamine-related variables and placebo responses, understanding such variables in depression may help tailor interventions more effectively.^31^ However, while most existing studies have focused on pain,^32^ research on antidepressant placebo responses is scarce.^33^

If and how individual differences in dopamine-related traits moderate antidepressant placebo responses are not clear, and competing hypotheses can be formulated. The placebo-reward hypothesis postulates that dopaminergic responsiveness may be crucial for placebo responses.^20^ Linked to reduced dopamine functioning, high anhedonia might thus impede symptom improvement via placebo,^34^ aligning with findings that traits negatively correlated to anhedonia (eg, optimism and extraversion) predict stronger placebo responses.^26^^,^^35^ On the other hand, positive treatment expectations may particularly enhance dopamine processing in individuals with high vs. low anhedonia, since expecting an increase in positive affect may be more rewarding to those with low positive affect/high anhedonia to begin with. Supporting this hypothesis, antidepressant treatment expectations have been found to reduce depressiveness-induced cardiac slowing in high anhedonia among healthy individuals.^36^ Furthermore, lower optimism (linked to high anhedonia)^37^ has been shown to predict better placebo treatment against stress in a healthy sample.^38^ Thus, high anhedonia may predict either weaker or stronger placebo responses. However, while placebo responses among healthy individuals have been frequently reported in the context of various disorders,^22^^,^^23^^,^^25^^,^^26^^,^^28^^,^^30^^,^^35^^,^^36^^,^^38^ research directly linking dopamine-related traits to antidepressant placebo responses is sparse.^36^

Depressive disorders involve dysfunctional affective experiences that come along with substantial limitations in well-being and daily functioning, posing a significant challenge in identifying and understanding successful treatment approaches. In order to gain insight into fundamental mechanisms and facilitate their translation into clinical applications, it is essential to examine specific dimensions of affective experiences (eg, positive affect and dopaminergic functioning) in nonclinical individuals, given the potential for subclinical symptoms to evolve into clinical disorders. As such, the present study investigated the role of depression- and dopamine-related traits and dopamine in antidepressant placebo responses using a randomized, double-blind, placebo-controlled 2 × 2 design with pharmacological (inert pills or dopamine D2 receptor antagonist sulpiride (400 mg)) and expectations (labels of either inactive or antidepressant) manipulations in *N* = 297 healthy individuals. We hypothesized that antidepressant treatment expectations would enhance state positive affect. Additionally, we hypothesized that these treatment expectation effects would involve the dopamine system, and accordingly be altered in the sulpiride vs. placebo substance group. We further assumed that treatment expectations effects would vary across individuals as a function of dopamine- and depression-related traits. As competing hypotheses, we specifically tested that higher anhedonia would relate to higher treatment expectation (ie, placebo) effects,^cf.^^36^ or to lower treatment expectation effects.^cf.^^26^ Finally, based on models linking trait anhedonia and extraversion to state positive affect via dopaminergic mechanisms, we explored whether sulpiride would alter the correlation between trait anhedonia (and extraversion) and state positive affect.

## METHODS

### Sample

A total of *N* = 297 healthy individuals (18-60 years, right-handed, German native speakers) participated in the study. Eligibility was determined through self-reports in a telephone interview. Exclusion criteria included: current psychiatric, neurological, autoimmune, hormonal, or cardiovascular conditions; any recent prescription medication use (past 3 months); pregnancy or hormonal contraception; liver, kidney, or bowel disorders; allergy to sulpiride, lactose, fructose, or gluten; regular smoking (>1/week); alcohol or illegal substance abuse; excessive caffeine intake (>8 cups/day); BMI < 19 or > 30. Informed consent was obtained prior to participation. The study, including the use of authorized deception, was approved by the Ethics Committee of Marburg University’s Medical Department, following the Declaration of Helsinki.

Two participants were excluded prior to analysis due to abnormal prolactin levels (see Supplementary Material), and 2 more due to missing baseline anhedonia scores, resulting in a final sample size of *N* = 293 (147 females; age: M = 25.13 years, SD = 4.2, range: 20-60). Group allocation was: *n* = 73 (no-substance expectation//placebo), *n* = 74 (no-substance expectation//sulpiride), *n* = 72 (antidepressant expectation//placebo), *n* = 74 (antidepressant expectation//sulpiride).

### Procedure

A detailed description of the entire procedure is included in the Supplementary Material.

#### Procedure for the Experimental Session

Participants arrived at 8 am and provided a baseline blood sample (8 mL) to assess plasma prolactin levels, which were also measured after substance intake to test for drug response^39^^,^^40^ (see Supplementary Material for prolactin analyses). Participants were then administered 2 identical capsules along with standardized verbal instructions (see Supplementary Material) manipulating treatment expectations. To induce antidepressant expectations, participants were told the capsules contained sulpiride, which would cause short-term mood enhancements noticeable after about 3 h, even in individuals without depression. For no-substance expectations, the capsules were stated as inactive. After receiving the instructions, participants swallowed the capsules.

Regardless of expectations, either sulpiride (2 × 200 = 400 mg; Neuraxpharm, Germany) or placebo pills (Neuraxpharm, Germany) were administered, resulting in a 2 × 2 design with Expectation (antidepressant vs no-substance) and Substance (placebo vs sulpiride); both sulpiride and placebo capsules were visually identical. Group allocation followed a randomized, double-blind protocol. After pill intake, participants received a standardized vegan breakfast.

One hour after intake, the second blood sample (8 mL) was obtained. Approximately 2 h and 45 min after intake, participants completed a 10-min resting phase followed by 3 computer tasks: a probabilistic selection task,^41^ an effort-based decision-making task,^42^ and a musical mood induction procedure.^36^ Before each task, participants were asked to complete a side effect questionnaire and indicate their treatment group. This was done to subtly reactivate the expectation manipulation throughout the session. Throughout the tasks, participants rated their affective states. At the session’s end, participants reported which substance they believed to have received and rated their certainty on a scale of 0 = *placebo* to 10 = *sulpiride* (Table S2). All participants were then fully debriefed about the nature and purpose of the study, including any use of deception. The study was conducted in German and analyses of individual tasks including the mood induction procedure were preregistered at ClinicalTrials.gov; ID: NCT05208294 and will be reported elsewhere. In the current report, we present analyses on the entire experimental session which had not been preregistered.

#### Substance

Sulpiride is a selective dopamine D2 receptor antagonist generally well tolerated with a low affinity for histaminergic, cholinergic, serotonergic, adrenergic, or GABA receptors. Slowly absorbed from the gastrointestinal tract, sulpiride reaches peak serum levels approximately 3 h after intake. Its elimination half-life averages 3 to 10 h.^43^ At low doses (50-200 mg), sulpiride presumably blocks presynaptic autoreceptors, elevating dopamine levels^44^ and reducing depressive symptoms, while higher doses predominantly block postsynaptic receptors. Doses up to 800 mg induce minimal side effects, allowing blinded group allocation.^45^ Here, 400 mg was employed, which should be sufficient to modulate dopaminergic processing^39^ with minimal side effect risk.

### Questionnaire Measures

#### Anhedonia

Within 2 days before the experiment, participants filled out online questionnaires including demographic data and trait measures. Trait anhedonia was assessed with a German adaptation of the 30-item Mood and Anxiety Symptom Questionnaire^46^ (MASQ-D30),^47^ which represents the tripartite model of mood^48^ and contains General Distress, Anhedonic Depression, and Anxious Arousal scales. On a 5-point Likert scale (1 = *not at all* to 5 = *extremely*), the 10-item Anhedonic Depression scale measures lack of Positive Affect with items like “Felt really happy” and “Felt like I had a lot of energy.” Higher reversed sum scores indicate higher Anhedonia, with excellent internal consistency in the present sample (Cronbach’s *α* = 0.91).

Anhedonia was also assessed via the German version^49^ of the Snaith-Hamilton Pleasure Scale (SHAPS).^50^ The internal consistency in the present healthy sample was *α* = 0.68. Here, we report the measure with the higher internal consistency, ie, MASQ-D30, as the primary measure of trait anhedonia. For comparability with other research, results of the SHAPS are also provided in the Supplementary Material.

#### Extraversion

After breakfast on the testing day, participants completed the German^51^ Big Five Aspect Scales (BFAS),^52^ including a 20-item measure of Extraversion. Higher scores indicate higher Extraversion. Internal consistency was high (*α* = 0.88). The Enthusiasm and Assertiveness facets were also computed for exploratory factor analysis (EFA) (see below).

#### Other Related Constructs

Additionally, participants completed several other questionnaires assessing relevant constructs including: the German^53^ revised Beck-Depression-Inventory (BDI-II),^54^ the German^55^ Temporal Experience of Pleasure Scale (TEPS),^56^ the German^57^ Life Orientation Test-Revised (LOT-R),^58^ the behavioral approach system (BAS) scales of the German^59^ Reinforcement Sensitivity Theory of Personality Questionnaire (RST-PQ) (see Supplementary Material for additional information),^60^ the BAS scales of the German^61^ Behavioral Inhibition System/Behavioral Activation System Scales (BIS/BAS),^62^ and a German Positive Valence Systems Scale (PVSS; own translation).^63^

### State Positive Affect

**Figure 1:**
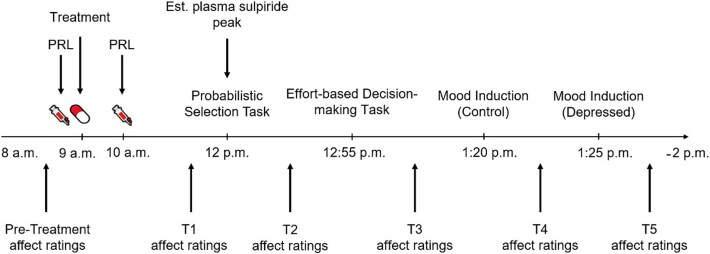
Sequential illustration of treatment, computer tasks and state positive affect ratings. Plasma peak of sulpiride was estimated to occur at approximately 3 h after intake (12 pm) when participants underwent the computer tasks. Abbreviations: Est., estimated; PRL, prolactin.

Participants rated their current affective states via the German^64^ Positive and Negative Affect Schedule (PANAS)^65^ before substance intake (pretreatment). The PANAS included 20 items, with 10 each assessing Positive (eg, “active,” “interested”) and Negative Affect on a 5-point Likert scale (1 = *not at all*, 5 = *extremely*). Before (T1) and at 4 subsequent time points during tasks and mood induction phases (T2-T5), participants repeated these ratings, resulting in 6 time points in total (Figure 1).

Given its relevance to anhedonia in depression, state positive affect assessed via PANAS is reported here as the primary outcome measure. Results of PANAS Negative Affect and additional mood ratings (eg, happiness, sadness; T1-T5) are included in the Supplementary Material.

### Statistical Analyses

All analyses were performed using R (v4.2.3) in RStudio.^66^ Linear mixed-effects models were fitted using the lmer function of the lmerTest package,^67^^,^^68^ with Time, Expectation, Substance, and Trait as fixed effects, and Subject as a random intercept. Omnibus tests (ie, ANOVAs) were conducted on the fitted models using the following specification:


\begin{align*} \mathrm{State}\ \mathrm{Positive}\ \mathrm{Affect}\sim &\ \mathrm{Time}\ast \mathrm{Expectation}\, \ast\\&\ \mathrm{Substance}\ast \mathrm{Trait}+\left(1|\mathrm{Subject}\right). \end{align*}

To examine whether substantial covariance among anhedonia, extraversion, and constructs related to dopamine and depression could be captured by any underlying factors associated with placebo responses, an EFA was conducted (jmv package’s efa function) with Minimum Residuals and oblimin rotation. Eigenvalues >1 were used for extraction. Factor scores were calculated with Thurstone estimation and included as a *z*-standardized continuous variable in the linear mixed-effects model. The following scales were included in the EFA: MASQ-D30 Anhedonic Depression, SHAPS-D, BFAS-Extraversion, BDI-II, TEPS, LOT-R, RSTPQ-BAS, BIS/BAS, and PVSS (see *Other Related Constructs* and *Factor Extracted from EFA*).

## RESULTS

Baseline anhedonia scores in the current sample, averaged across all groups, were comparable with a healthy sample in our previous study, which demonstrated antidepressant placebo responses among participants high in anhedonia (see Supplementary Material with regard to restricted variability).^36^ Separate ANOVAs including experimental conditions as factor confirmed that baseline traits scores and age did not differ across groups (Table 1). Descriptive statistics including mean, standard deviation, and range across all administered questionnaires are reported in Table S1. There were no significant between-group differences across these measures (all *P* > .29).

**Table 1:** Demographic characteristics at baseline.

	NS//PLC		NS//SUL		AD//PLC		AD//SUL		Full sample		
Baseline characteristic	*n*	%	*n*	%	*n*	%	*n*	%	*n*	%	
**Female sex**	36	49.3	37	50	37	51.3	37	50	147	50.2	

	M	SD	M	SD	M	SD	M	SD	M	SD	*P*
**Age**	25.5	4.0	25.0	5.5	25.4	3.2	24.5	3.5	25.1	4.2	.46
**MASQ-D30 Anhedonia**	26.3	7.5	27.1	7.1	26.7	7.7	27.0	7.4	26.8	7.4	.93

Abbreviations: AD, antidepressant expectation; NS, no-substance expectation; PLC, placebo substance; SUL, sulpiride substance.

Participants’ sex included either female or male.

### Manipulation Check

#### Substance Manipulation

A Substance × Expectation ANOVA on participants’ plasma prolactin change confirmed the expected main effect of Substance (*F*(1, 193) = 269.97, *P* < .001), such that the placebo group had a smaller change (M = −1.18, SD = 2.00) than the sulpiride group (M = 62.50, SD = 38.0), *t*(99) = −16.72, *P* < .001. Expectation did not affect prolactin levels (*F*(1, 193) = 2.04, *P* = .155); plasma prolactin levels for all participants are plotted in Figure S1.

We additionally tested the associations between sex, body weight, and prolactin change and found a larger prolactin increase in females compared to males (*P* < .001; see Supplementary Material). Finally, testing whether sulpiride-induced changes in plasma prolactin were associated with trait anhedonia and extraversion^9^ revealed no significant associations (see Supplementary Material).

#### Expectation Manipulation

A Substance × Expectation ANOVA on the self-rated belief to have received inert pills vs sulpiride confirmed a significant main effect of Expectation (*F*(1, 495) = 86.39, *P* < .001), such that participants in the antidepressant expectation group were more likely to believe that they had received sulpiride than the no-substance expectation group.

Additionally, both Expectation (*P* = .670) and Substance (*P* = .220) manipulation did not predict posttreatment self-reported side effects (see Supplementary Material).

### State Positive Affect over Time

#### Anhedonia

The omnibus test of the model on positive affect ratings revealed a main effect of Time (*F*(5, 1419) = 84.07, *P* < .001, *η*^2^_p_ = .229), indicating that positive affect varied significantly across time points. Estimated marginal means (EMM) revealed that state positive affect decreased from pretreatment (EMM = 2.72, SE = 0.04) to T1 (EMM = 2.42, SE = 0.04), then gradually increased throughout T2 (EMM = 2.57, SE = 0.04), peaking at T3 (EMM = 2.76, SE = 0.04), and subsequently declined at T4 (EMM = 2.53, SE = 0.04), with the lowest during T5 (EMM = 2.06, SE = 0.04) (see Supplementary Material). There was also a main effect of Anhedonia (*F*(1, 285) = 21.37, *P* < .001, *η*^2^_p_ = .070) indicating lower state positive affect in high anhedonia. In contrast to our hypotheses, no main effects of Expectation and Substance, and no Expectation × Substance interaction were observed across the sample (*P* > .273). However, we observed a Substance × Anhedonia interaction (*F*(1, 285) = 7.19, *P* = .008, *η*^2^_p_ = .025) further qualified by a Substance × Anhedonia × Time interaction, *F*(5, 1419) = 2.23, *P* = .049, *η*^2^_p_ = .008.

**Figure 2:**
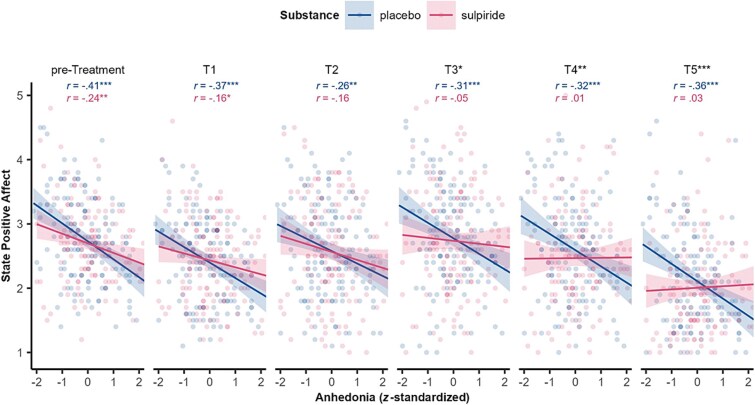
State positive affect via PANAS throughout the experimental session contrasted with *z*-standardized baseline trait anhedonia score and separated for substance groups. Black asterisks indicate significantly different correlations. ^*^P < .05; ^**^P < .01; ^***^P < .001.

To further investigate this 3-way interaction, follow-up Pearson correlations between anhedonia and state positive affect were computed for each time point and substance group. Pretreatment, there was an expected negative association for both groups, such that lower positive affect ratings were associated with higher anhedonia (placebo: *r*(141) = −0.41, *P* < .001; sulpiride: *r*(143) = −0.24, *P* = .004; *Z* = −1.65, *P* = .098; Figure 2). This negative association persisted under placebo. Under sulpiride, however, it decreased from pretreatment (*r* = −0.24, *P* < .05) over T1 (approximately 3 h postintake; *r* = −0.16, *P* = .050) to T2 (*r* = −0.16, *P* = .060), T3 (*r* = −0.05, *P* = .512), T4 (*r* = 0.01, *P* = .939), and T5 (*r* = 0.03, *P* = .685). Fisher’s *Z*-tests revealed that significant correlation differences between substance groups emerged at T3 (*Z* = −2.25, *P* = .025) and persisted throughout T4 (*Z* = −2.86, *P* = .004) and T5 (*Z* = −3.51, *P* < .001), while they were absent before substance intake (pretreatment: *Z* = −1.65, *P* = .098) and shortly after (T1: *Z* = −1.91, *P* = .056) and T2 (*Z* = −0.93, *P* = .353; Figure 2). No other effects emerged (all *P* > .192; Table S3). Similar patterns were observed for SHAPS-D and BDI-II (see Supplementary Material).

To test the specificity of Anhedonia, separate omnibus tests on the models were additionally conducted with MASQ-D30 Anxious Arousal and General Distress subscales in place of Anhedonia scores. These models did not yield similar results (see Supplementary Material).

#### Extraversion

**Figure 3:**
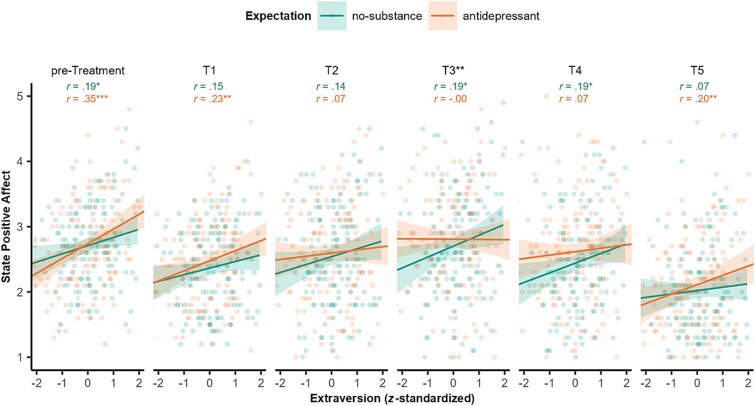
State positive affect via PANAS throughout the experimental session contrasted with *z*-standardized baseline trait extraversion score and separated for expectation groups. Black asterisks indicate significantly different correlations. ^*^P < .05; ^**^P < .01; ^***^P < .001.

In line with prior research,^69–71^ anhedonia and extraversion were negatively correlated in the present sample (*r*(290) = −0.54, *P* < .001). Given its negative association with anhedonia^69–71^ and positive association with positive affect,^72^ we tested whether an omnibus test on the model with Extraversion as z-standardized continuous variable would reveal comparable effects to Anhedonia. The omnibus test revealed main effects of Time (*F*(5, 1415) = 82.66, *P* < .001, *η*^2^_p_ = .226) and Extraversion (*F*(1, 284) = 10.05, *P* = .002, *η*^2^_p_ = .034), a trend Substance × Extraversion interaction (*F*(1, 284) = 3.73, *P* = .054, *η*^2^_p_ = .013), and a Time × Expectation × Extraversion interaction (*F*(5, 1415) = 3.68, *P* = .003, *η*^2^_p_ = .013). The 3-way interaction indicated that the expected positive association between Extraversion and positive affect which was observed pretreatment (*r*(286) = 0.27, *P* < .001) only persisted under no-substance expectations, but diminished from T2 to T4 due to relative increases in introverts’ positive affect under antidepressant vs. no-substance expectations (Figure 3). Thus, in line with our hypothesis, antidepressant treatment expectations raised positive affect for introverts but not for extraverts.

Although the Substance × Extraversion interaction was not significant here, we explored whether the association between Extraversion and positive affect over time indicated a similar susceptibility to the pharmacological manipulation as Anhedonia. As shown in the Supplementary Material, a comparable result pattern was observed.

#### Factor Extracted from EFA

The EFA of 16 different anhedonia and extraversion scales revealed 1 factor with Eigenvalue >1 (4.830; subsequent Eigenvalues: 0.881, 0.757), which we term Positive Affectivity. Factor loadings are summarized in Table 2. The omnibus test on the model revealed main effects of Time (*F*(5, 1398) = 87.41, *P* < .001, *η*^2^_p_ = .238) and Positive Affectivity (*F*(1, 280) = 20.20, *P* < .001, *η*^2^_p_ = .067), a Substance × Positive Affectivity interaction (*F*(1, 280) = 7.68, *P* = .006, *η*^2^_p_ = .027), and a Time × Expectation × Positive Affectivity interaction (*F*(5, 1398) = 2.34, *P* = .040, *η*^2^_p_ = .008). Like Anhedonia and Extraversion, Positive Affectivity was positively associated with positive affect across substance groups pretreatment, *r*(285) = 0.35, *P* < .001. Posttreatment (ie, for T1-T4), this association persisted over time for placebo (*r*(142) = 0.36, *P* < .001) but not sulpiride (*r*(141) = 0.09, *P* = .147; *Z* = 2.39) (Figure S2). Moreover, the 3-way interaction indicated that this positive association persisted from pretreatment to T4 under no-substance expectations (*r* = 0.29), but diminished at T3 under antidepressant expectations (*r* = 0.07; *Z* = 1.87) (Figure S3). Thus, the correlation between a general trait Positive Affectivity factor and state positive affect ratings was initially present in the entire sample but then disrupted by both sulpiride and antidepressant treatment expectations. Correlation Coefficients for all questionnaires included in the EFA are included in Table S4. No further effects emerged (all *P* > .096).

**Table 2:** Results from the EFA of the related constructs

Scale	Factor loading
**MASQ-D30 Anhedonic Depression**	−0.73
**SHAPS-D**	−0.33
**BFAS-Extraversion**	
** Enthusiasm**	0.71
** Assertiveness**	0.55
**BDI-II**	−0.50
**TEPS**	
** Consummatory Pleasure**	0.36
** Anticipatory Pleasure**	0.46
**LOT-R**	0.57
**RSTPQ-BAS**	
** Reward Interest**	0.72
** Goal-Drive Persistence**	0.52
** Reward Reactivity**	0.69
** Impulsivity**	0.47
**BIS/BAS**	
** Drive**	0.54
** Fun Seeking**	0.47
** Reward Responsiveness**	0.69
**PVSS**	0.50

## DISCUSSION

This study sought to examine the complex interplay of dopamine, expectations, and positive affect-related personality traits on state positive affect. In a 2 × 2 placebo-controlled design involving pharmacological and expectation manipulation in a large sample, we found that the effects of the experimental treatment expectation manipulation and sulpiride crucially depended on individual differences in Extraversion and Anhedonia, respectively, or, more generally, on a broad Positive Affectivity factor. Contrary to our expectations, no main effects of treatment expectation or sulpiride were observed. The observed interactions indicate that antidepressant treatment expectations and sulpiride particularly raise state positive affect in individuals with low positive affective traits.

### Antidepressant Treatment Expectation Effects in Low Positive Affectivity

Antidepressant treatment expectations did not enhance state positive affect across the board as we hypothesized. Rather, antidepressant treatment expectations increased state positive affect among introverts during T2-T4, as evidenced in a disruption of the prototypical correlation of extraversion and state positive affect during these time windows. A similar pattern (albeit non-significant) emerged for anhedonia, such that its negative association with state positive affect decreased at T3 under antidepressant expectations. Finally, a trait × Expectation interaction also emerged for the broad Positive Affectivity factor that captured the covariance of various extraversion and anhedonia scales. Initially, Positive Affectivity was correlated with state positive affect, but antidepressant treatment expectations selectively enhanced state positive affect in individuals with low Positive Affectivity. These observations align with our previous findings that antidepressant expectations attenuated depressiveness-induced cardiac slowing in high vs. low anhedonia^36^ and support our hypothesis that higher anhedonia (or lower Positive Affectivity) facilitate antidepressant treatment expectation effects. They further converge with prior findings that lower extraversion predicted stronger placebo responses against stress,^38^ and that novelty seeking, an extraversion- and dopamine-related trait, was lower in individuals susceptible to placebo-induced sensations.^73^ At the same time, this group of results contrasts with studies suggesting that higher extraversion^26^^,^^27^ and optimism^25^ predict stronger placebo responses. Notably, these diverging findings mostly focused on pain rather than state positive affect. Thus, optimism may facilitate placebo analgesia but may not generalize to depression-related placebo responses, in which lower levels of positive affect may be necessary to motivate mood enhancements. Aligning with the association between low extraversion and depressive symptoms (ie, anhedonia),^69–71^ our findings demonstrate that dopamine- and depression-related traits moderate antidepressant placebo responses, which may hinge on depressiveness magnitude. Moreover, no expectation effects were revealed with anxiety-related scales (see Supplementary Material), underscoring the specificity of low positive affectivity. While domain-specific research remains inconclusive and scarce,^33^ our findings highlight the role of individual differences in antidepressant placebo responses, underscoring the importance to probe variables relevant to depression.

### Effects of Dopaminergic Substance Parallel Treatment Expectation

Sulpiride increased state positive affect in participants with high vs. low anhedonia. Similar patterns emerged for extraversion and Positive Affectivity, such that sulpiride raised introverts’ lower state positive affect, while reducing extraverts’ higher baseline positive affect. Likewise, there was a positive association between Positive Affectivity and state positive affect before treatment, which was disrupted by sulpiride: state positive affect was elevated among participants with lower Positive Affectivity, whereas it was decreased in higher levels.

Our results suggest that sulpiride may have an equally-breaking effect on state positive affect, ie, increasing in individuals with higher anhedonia, while decreasing in lower anhedonia. This aligns with prior research indicating paradoxical (U-shaped) effects of dopamine manipulation depending on baseline characteristics. eg,^11^^,^^74^^,^^75^ While its underlying mechanism remains debated, eg,^76–78^ sulpiride may enhance mood in high anhedonic individuals by compensating for lower baseline dopamine signaling. Conversely, individuals with lower anhedonia and intact dopamine functioning may experience reduced positive affect due to sulpiride’s postsynaptic action, which presumably reduced dopamine signaling. This effect may be smaller in high anhedonia due to relative blunted baseline responsiveness. Accordingly, sulpiride has been shown to produce antidepressant effects in mild to moderate depression^16^ and increase positive affect among introverts.^11^ Moreover, 400 mg sulpiride has been reported to enhance motivation specifically in low dopamine synthesis capacity.^76^ While another study reported attenuated hedonic responses to pleasant stimuli following D2 receptor antagonist intake, baseline traits were not considered.^79^

To some degree, the observed pharmacological effects parallel the previously discussed antidepressant expectation effects: both manipulations disrupted the correlations between state positive affect and extraversion, anhedonia, and Positive Affectivity. The similarity of these patterns provides support for the assumption that treatment expectation effects involve the dopamine system and are altered under sulpiride, ie, dopamine manipulation enhanced state positive affect in participants with relative high anhedonia levels, while producing contrasting effects in lower anhedonia levels. We speculate that high anhedonia is related to relative lower dopamine sensitivity, whereas individuals with low anhedonia have relatively higher dopamine signaling. Moreover, our results are consistent with the notion that the link between dopamine and anhedonia is not limited to the motivational component but may also involve the pleasure-related facet of anhedonia, as indicated by a converging result pattern when the Consummatory Pleasure of the TEPS, a scale presumably reflecting pleasure aspects of anhedonia, was analyzed (see Supplementary Material). In sum, our findings suggest that individual differences in dopaminergic functioning modulate antidepressant placebo responses, eg,^22^^,^^23^ and contribute to research on neurobiological mechanisms underlying such responses.

Interestingly, however, no significant interaction between substance and expectation manipulation was observed. If placebo responses were driven by dopamine, the expectation manipulation effects may have been disrupted by sulpiride as hypothesized, especially given the presumably high dosage of 400 mg. As this was not the case, a possible interpretation is that sulpiride acted not only as an antagonist via postsynaptic blockade among all participants, but may also have exhibited agonist-like effects through blocking presynaptic autoreceptors.^cf.^^76^ Additionally, while both dopaminergic and expectation manipulation increased state positive affect in individuals with low Positive Affectivity, they may rely on only partially overlapping neural systems (eg, involved in more subtle experience vs. more explicit ratings of affect, respectively), allowing their effects to remain independent to some extent.

### Implications

Previous research has shown substantial evidence for expectation-induced placebo responses in both healthy and depressed participants. eg,^80–82^ A recent meta-analysis further confirmed consistent effects across treatment modalities.^83^ However, most evidence emerges from clinical settings and centers on pain.^84^ Understanding whether antidepressant placebo responses differ between healthy and clinically diagnosed individuals remains limited. Our study, employing a pharmacological challenge in a large, healthy sample, demonstrates that such responses may hinge on depressiveness magnitude and the presence of depressive experience. Additionally, the effects observed in the present study are specific to positive affect and do not emerge for negative affect (see Supplementary Material). While most studies focus on negative affective experiences,^81^^,^^82^^,^^85^^,^^86^ targeting positive affect may be particularly relevant for anhedonia and reward hyposensitivity as central aspects of depression.^36^^,^^87^^,^^88^

Limited research has specifically examined the link between dopamine functioning and affective experience, and existing studies rarely assess relevant baseline traits.^79^^,^^89–91^ Our study offers valuable insights into how individual differences in these traits moderate dopaminergic drugs effects on mood.^92^ Furthermore, the observed pattern for Positive Affectivity reflects the effects of both treatment expectations and sulpiride, supporting our assumption that anhedonia, depression, and dopamine functioning are key factors in these responses.

### Limitations and Conclusions

Antidepressant placebo and substance responses were observed only among participants with lower Positive Affectivity (ie, lower extraversion/higher anhedonia). While we interpret this as highlighting the role of individual differences, an alternative interpretation is that there may have been ceiling effects such that high treatment expectations and/or sulpiride could not further enhance positive affect in healthy individuals with higher Positive Affectivity. However, this interpretation would be at odds with the observation that sulpiride and antidepressant treatment expectations tended to decrease (rather than maintain) state positive affect in low anhedonia and high extraversion, respectively. A second limitation may be that the experimental expectation manipulation was not sufficiently convincing for all participants, especially in a university setting where healthy participants were familiar with such setups. However, manipulation checks confirmed the effectiveness of the manipulation at the group level, and control analyses excluding participants who did not believe the instructed treatment yielded comparable results (see Supplementary Material). Thus, these results support our interpretation that a certain depressiveness magnitude is required for consistent antidepressant placebo responses in healthy participants.^36^ Future studies could explore this mechanism in more clinically diverse populations, allowing direct comparisons between healthy and diagnosed individuals.

This study is among the first to investigate how depression- and dopamine-related traits moderate antidepressant placebo responses, employing a pharmacological challenge in a large, healthy sample. Our findings indicate that low dispositional positive affectivity may be necessary for robust antidepressant placebo responses. Additionally, while dopamine functioning is essential for the underlying psychopharmacological mechanisms, baseline traits may influence the effects of dopamine antagonists. Taken together, our study highlights the weight of individual differences in both therapeutic and pharmacological approaches to depression treatment. Future research should consider these factors to develop more effective, tailored interventions.

## Supplementary Material

Supplemental_Material_revised_pyaf067_fin

## Data Availability

The data underlying this article can be made available upon reasonable request to the corresponding author.
