# Supplementary material for: Individual differences in dopamine-related traits influence mood effects of dopamine D2-antagonist and antidepressant treatment expectations
Source: Int J Neuropsychopharmacol. 2025 Sep 12;28(11):pyaf067. doi: 10.1093/ijnp/pyaf067 (PMC13223761; doi:10.1093/ijnp/pyaf067)
Supplement: Supplemental_Material_revised_pyaf067_fin [file supplemental_material_revised_pyaf067_fin.docx]

**Supplementary Material and Methods**

**Sample**

297 healthy individuals with normal or corrected-to-normal vision participated in the study for course credits (10 unit) or monetary compensation (130 EUR; participants were told they had the chance to earn 20 EUR extra which was stated to be the maximum reward attainable in the computer tasks). Participants were recruited via flyers, posters, personal advertisement, and the research participation system of the Psychology Department of Marburg University.

**Procedure**

Data were collected between December 2021 and September 2023. Participation consisted of two appointments: pre-testing pickup and the experimental session which lasted for approximately five to six hours.

***Procedure Prior to the Testing Session***

Upon interest in participation, individuals were interviewed by telephone to assess eligibility (see *Sample*). An additional exclusion criterion was prior knowledge of Japanese Hiragana characters used in one of the computer tasks. Written study information was sent to invited participants via mail before the experimental session, which stated that the study aimed to investigate the effect of dopamine on mood and learning processes, and that participants might receive either placebo or sulpiride (400 mg) capsules as part of the experimental design. The selective dopamine D2 receptor antagonist sulpiride was described as a certified medication that acts on dopamine receptors in the brain, and is prescribed as an antidepressant, among other uses, in clinical practice (for further information on sulpiride, see *Substance*). Two appointments were arranged for invited participants. For the pre-testing pickup, participants were given a saliva test to assess endocrine or genetic factors (concerning expectation effects) not relevant for the current analyses. Additionally, participants provided informed consent and were given the opportunity to clarify questions.

Participants were asked to get sufficient sleep and not to consume alcohol or eat after 10 p.m. on the previous day of the experiment. In addition, participants were reminded not to exercise and not to eat breakfast on the day of testing.

***Procedure for the Experimental Session***

Participants were greeted by the experimenter at 8 a.m. before they completed a COVID test. All female participants then concluded a pregnancy test. Participants then provided a saliva sample not relevant for the current report before the first blood sample (8 ml) for baseline prolactin assessment.

For the substance administration, the capsules were placed in a white envelope which was handed to participants by our medical staff. After receiving verbal instructions, participants were asked to take the pills orally.

After providing the second blood sample (8 ml) for peak plasma prolactin assessment (approximately one hour after substance intake), participants were then provided with magazines with neutral content to read and completed two rounds of the Alternative Uses Test^1^ not analyzed in the present report before electrodes for EEG and ECG recordings, also not analyzed in the present report, were attached. Approximately 2 hours and 45 minutes after substance intake, participants underwent a 10-minute resting phase, in which they were instructed to look at a white fixation cross presented in the center of the computer screen. Subsequently, participants completed three computer tasks. After the last computer task (i.e., the mood induction procedure), electrodes were removed from participants, and they filled out questionnaires described elsewhere^2^ and some questions about their experiences during the experiment.

***Standardized Verbal Instructions***

As part of the treatment expectation manipulation, standardized verbal instructions were employed for each expectation group. The original scripts, in German, translate as follows:

**Antidepressant Expectations.** *“You have been assigned to the medication group through a random selection and not to the placebo group. This means that you will receive a very effective medication, which should have a positive impact on your mood during the study. The medication is Sulpiride in a dose of 400 mg. Sulpiride is a medication approved in Germany and belongs to the group of antidepressants. It is used, for example, in people who suffer from depression. Even a single tablet can significantly improve mood. This means that one will feel much better and experience more joy. The special thing is that the mood-lifting effect even develops in individuals who do not suffer from depression. Sulpiride is a very safe and well-tolerated medication. Like any other medication, it can rarely cause side effects. If you experience any discomfort, please let us know.*

*It takes about three hours for Sulpiride to reach its full effect in the brain. You should then notice that your mood improves significantly during the experiment. If you have any questions, please feel free to ask them at any time.”*

**No-Substance Expectations. *“****You have been assigned to the placebo group through a random selection and not to the medication group. This means that you will receive placebo capsules shortly, which do not contain any active ingredients and therefore will not have any effects on your body during the study. If you have any questions, please feel free to ask them at any time.”*

**Plasma Prolactin Measures**

Plasma prolactin levels were measured at baseline and one hour after substance intake (Figure 1) using an ELISA kit (IBL International), a solid phase enzyme-linked immunoassay (Intra-/inter-assay: 2.91-5.87/5.64-6.22%; detection limit: 0.35 ng/ml). For prolactin analyses, data for both time points were available for *n* = 202 due to missing data and staff unavailability. Two were excluded due to abnormal levels, resulting in n = 200 (Figure S1).

**Supplementary Results**

**Manipulation Check**

***Substance Manipulation***

Participants’ plasma prolactin levels are shown in Figure S1. Two participants in the antidepressant expectation group were excluded from further analyses due to abnormal levels: One received placebo (participant *z*-score: 6.59), another received sulpiride (participant z-score: 6.17). Data removal did not alter the result pattern.

**Effects of Sex and Body Weight.** We tested the association between sex, body weight, and plasma prolactin change and found a larger increase in females (*F*(1, 193) = 19.54, *p* < .001). Specifically, the change between time points was larger for females (*M* = 44.03, *SD* = 51.8) vs. males (*M* = 18.90, *SD* = 24.0; *t*(132) = 4.33, *p* < .001).

**Associations between traits and Plasma Prolactin Change.** We tested whether sulpiride-induced changes in plasma prolactin were associated with trait anhedonia and extraversion, respectively. Pearson correlations between prolactin change (z-standardized by sex) and traits were computed for each substance. For placebo, no significant associations were found with anhedonia (*r*(95) = 0.14, *p* = .186) or extraversion (*r*(95) = -0.15, *p* = .150). Similarly, for sulpiride, no significant associations were observed with anhedonia (*r*(98) = -0.04, *p* = .693) or extraversion (*r*(97) = -0.05, *p* = .608).

**Side effects.** Side effects were assessed after the intervention procedure using the General Assessment of Side Effects questionnaire (GASE)^3^. Neither the expectation (*p* = .670) nor the substance (*p* = .220) manipulation predicted self-reported side effects.

***Expectation Manipulation***

At the end of the experimental session, participants indicated which treatment they believed they had received on a scale of 0 (*placebo*) to 10 (*sulpiride*). These ratings are summarized in supplemental Table S2. In addition, control analyses of the current results were computed with participants who indicated to believe that they had received the instructed treatment. To this end, participants with a rating of less than 5 were categorized as believing they had received placebo, while those with a rating of 5 or higher were categorized as believing they had received sulpiride. The omnibus test with these participants (n = 180) yielded comparable results, revealing a main effect of Time (*F*(5, 856) = 40.13, *p* < .001, η^2^*_p_* = .190), a main effect of Anhedonia (*F*(1, 172) = 9.27, *p* = .003, η^2^*_p_* = .051), a trend for a Substance × Anhedonia interaction (*F*(1, 172) = 3.72, *p* = .056, η^2^*_p_* = .021), and a Time × Anhedonia interaction (*F*(5, 856) = 2.23, *p* = .020, η^2^*_p_* = .015). No additional significant effects were observed (all *p* > .097).

| **Figure S1**  *Plasma Prolactin at Baseline and One Hour after Treatment by Experimental Groups* |
| --- |
| 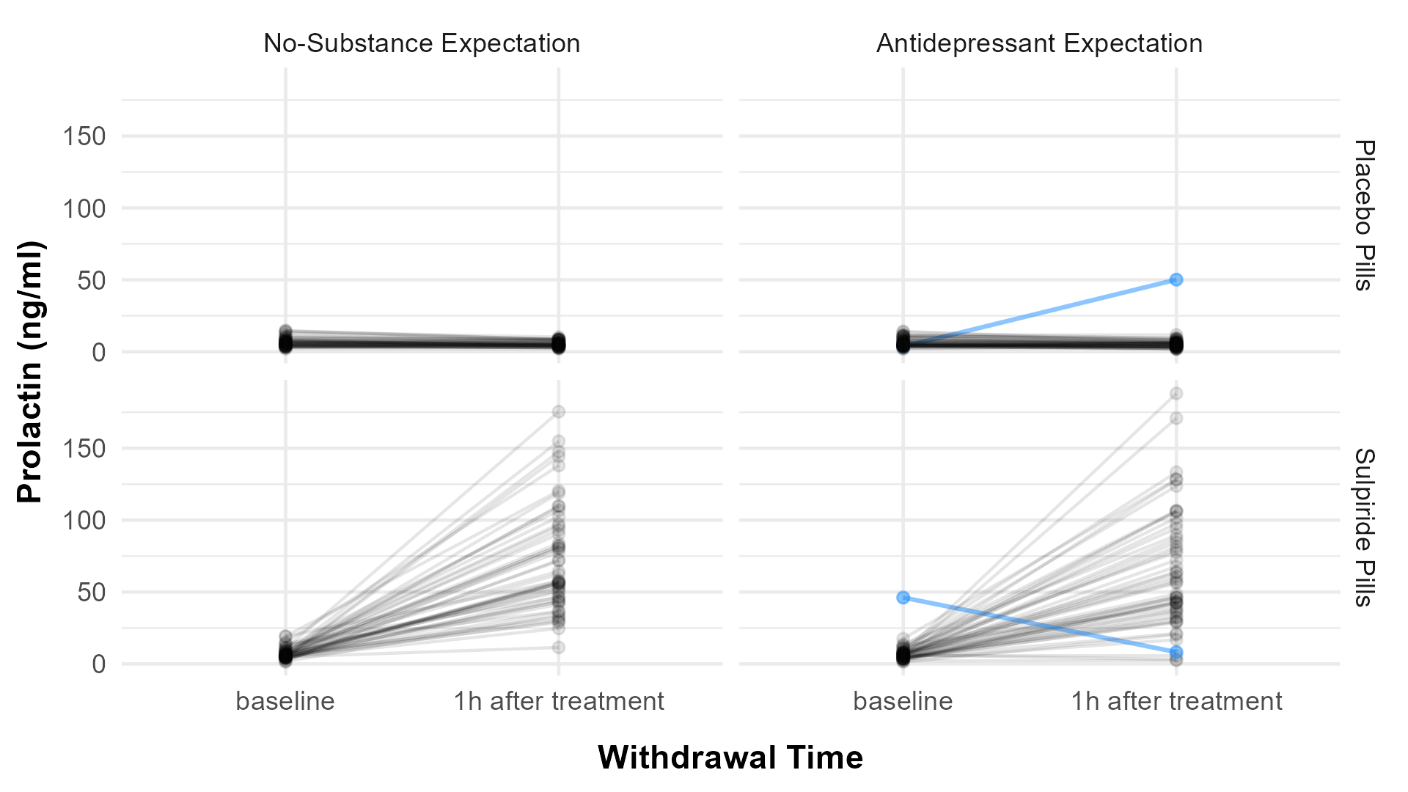 |
| *Note.* N = 202. Two participants were removed from further analyses (n = 200) due to abnormal plasma prolactin levels at baseline and one hour after treatment, as marked in blue. |

**Table S1**

*Demographic Characteristics and Trait Measures at Baseline*

| Baseline characteristic | NS//PLC | | NS//SUL | | AD//PLC | | | AD//SUL | | | Full sample | | |  |
| --- | --- | --- | --- | --- | --- | --- | --- | --- | --- | --- | --- | --- | --- | --- |
|  | n | % | n | % | | n | % | | n | % | | n | % |  |
| Female sex | 36 | 49.3 | 37 | 50 | | 37 | 51.3 | | 37 | 50 | | 147 | 50.2 |  |
|  | M (SD) | Range | M (SD) | Range | | M (SD) | Range | | M (SD) | Range | | M (SD) | Range | *p* |
| Age | 25.5 (4.0) | 20–41 | 25.0 (5.5) | 20–60 | | 25.4 (3.2) | 20–36 | | 24.5 (3.5) | 20–43 | | 25.1 (4.2) | 20–60 | .46 |
| MASQ-D30 AH | 26.3 (7.5) | 11–49 | 27.1 (7.1) | 12–46 | | 26.7 (7.7) | 13–47 | | 27.0 (7.4) | 12–43 | | 26.8 (7.4) | 11–49 | .93 |
| SHAPS-D AH | 0.76 (1.3) | 0–6 | 0.78 (1.3) | 0–5 | | 0.76 (1.0) | 0–8 | | 0.97 (1.8) | 0–7 | | 0.82 (1.4) | 0–8 | .38 |
| BFAS-Extraversion | 4.99 (0.8) | 2.6–6.4 | 4.94 (0.7) | 3.1–6.4 | | 4.85 (0.8) | 3.1–6.7 | | 4.79 (0.9) | 2.9–6.5 | | 4.89 (0.8) | 2.6–6.7 | .45 |
| BDI-II | 5.36 (4.7) | 0–19 | 5.66 (5.1) | 0–22 | | 6.13 (4.7) | 0–19 | | 6.38 (6.3) | 0–33 | | 5.88 (5.5) | 0–33 | .68 |
| TEPS | 4.56 (0.5) | 3.1–5.7 | 4.46 (0.5) | 3.1–5.4 | | 4.53 (0.6) | 2.9–5.9 | | 4.61 (0.5) | 3.1–5.7 | | 4.54 (0.5) | 2.9–5.9 | .29 |
| LOT-R | 17.62 (2.2) | 12–21 | 17.35 (2.4) | 11–21 | | 17.10 (2.6) | 10–21 | | 17.07 (2.3) | 11–21 | | 17.28 (2.4) | 10–21 | .46 |
| RSTPQ-BAS | 2.80 (0.4) | 1.9–3.6 | 2.73 (0.4) | 2.0–3.7 | | 2.82 (0.4) | 1.5–3.4 | | 2.76 (0.4) | 1.7–3.7 | | 2.78 (0.4) | 1.5–3.7 | .57 |
| BIS/BAS | 1.95 (0.3) | 1.1–2.8 | 2.01 (0.4) | 1.2–2.8 | | 1.90 (0.4) | 1.4–2.9 | | 1.94 (0.3) | 1.0–2.9 | | 1.95 (0.3) | 1.0–2.9 | .30 |
| PVSS | 7.04 (1.0) | 2.2–8.7 | 6.79 (1.2) | 1.9–8.5 | | 7.05 (1.1) | 1.5–8.5 | | 7.03 (0.8) | 3.8–8.3 | | 6.98 (1.0) | 1.5–8.7 | .36 |

*Note.* NS = no-substance expectation; AD = antidepressant expectation; PLC = placebo substance; SUL = sulpiride substance. AH = Anhedonia. Participants’ sex included either female or male.

**Table S2**

*Credibility Assessment on Expectation Manipulation by Experimental Group*

|  | NS//PLC | | NS//SUL | | | AD//PLC | | AD//SUL | | | | Full sample | |  |
| --- | --- | --- | --- | --- | --- | --- | --- | --- | --- | --- | --- | --- | --- | --- |
|  | n | *%* | n | *%* | n | | *%* | | n | *%* | n | | *%* |  |
| Rating < 5  Rating ≥ 5  No answer | 63  9  1 | 86.3  12.3  1.37 | 69  5  0 | 85.1  6.8  0 | 50  21  1 | | 69.4  29.2  1.4 | | 46  27  1 | 62.2  36.5  1.4 | 228  62  3 | | 77.8  21.2  1 |  |
|  | M | SD | M | SD | M | | SD | | M | SD | M | | SD | *p* |
|  | 1.4 | 2.0 | 1.2 | 1.8 | 3.7 | | 2.5 | | 4.2 | 3.0 | 2.6 | | 2.7 | < .001 |

*Note.* *N* = 290. Participants indicated the treatment they believed to have received after the experiment on a scale of 0 (*placebo*) to 10 (*sulpiride*). NS = no-substance expectation; AD = antidepressant expectation; PLC = placebo substance; SUL = sulpiride substance. Three participants did not complete the credibility assessment.

**State Positive Affect over Time**

The omnibus test of the model on positive affect ratings revealed a main effect of Time (*F*(5, 1419) = 84.07, *p* < .001, *η*^2^_p_ = .229), indicating that positive affect varied significantly across time points. To get a clearer overview of the direction of the changes, we calculated estimated marginal means (EMM) for each time point. Results revealed that state positive affect decreased from pre-Treatment (EMM = 2.72, SE = 0.04, 95% CI [2.64, 2.80]) to T1 (EMM = 2.42, SE = 0.04, 95% CI [2.34, 2.50]), then gradually increased throughout T2 (EMM = 2.57, SE = 0.04, 95% CI [2.49, 2.66]), peaking at T3 (EMM = 2.76, SE = 0.04, 95% CI [2.68, 2.84]), and subsequently declined at T4 (EMM = 2.53, SE = 0.04, 95% CI [2.45, 2.61]), with the lowest during T5 (EMM = 2.06, SE = 0.04, 95% CI [1.98, 2.14]).

**Table S3**

*Effects of Substance, Expectation, Time and Anhedonia on State Positive Affect*

| Effect | *F* (1, 285)  *F* (5, 1419) | *p* | η^2^*_p_* |
| --- | --- | --- | --- |
| Expectation (EX) | 1.21 | .273 | .002 |
| Substance (S) | 0.51 | .475 | .002 |
| Time (T) | 84.07^***^ | < .001 | .229 |
| Anhedonia (AH) | 21.37^***^ | < .001 | .070 |
| EX × S | 0.22 | .637 | .001 |
| EX × T | 0.99 | .421 | .003 |
| S × T | 1.14 | .338 | .004 |
| EX × AH | 0.28 | .596 | .001 |
| S × AH | 7.19^**^ | .008 | .025 |
| T × AH | 1.48 | .192 | .005 |
| EX × S × TP | 0.53 | .754 | .002 |
| EX × S × AH | 0.18 | .673 | .001 |
| EX × T × AH | 1.31 | .258 | .005 |
| S × T × AH | 2.23^*^ | .049 | .008 |
| EX × S × T × AH | 1.44 | .208 | .005 |

*Note. ^*^p < .05; ^**^p < .01;* ^***^*p* < .001. *F* (5, 1419) for Time.

**Figure S2**

*State Positive Affect throughout the Experimental Session Predicted by Substance Groups and Positive Affectivity*

***
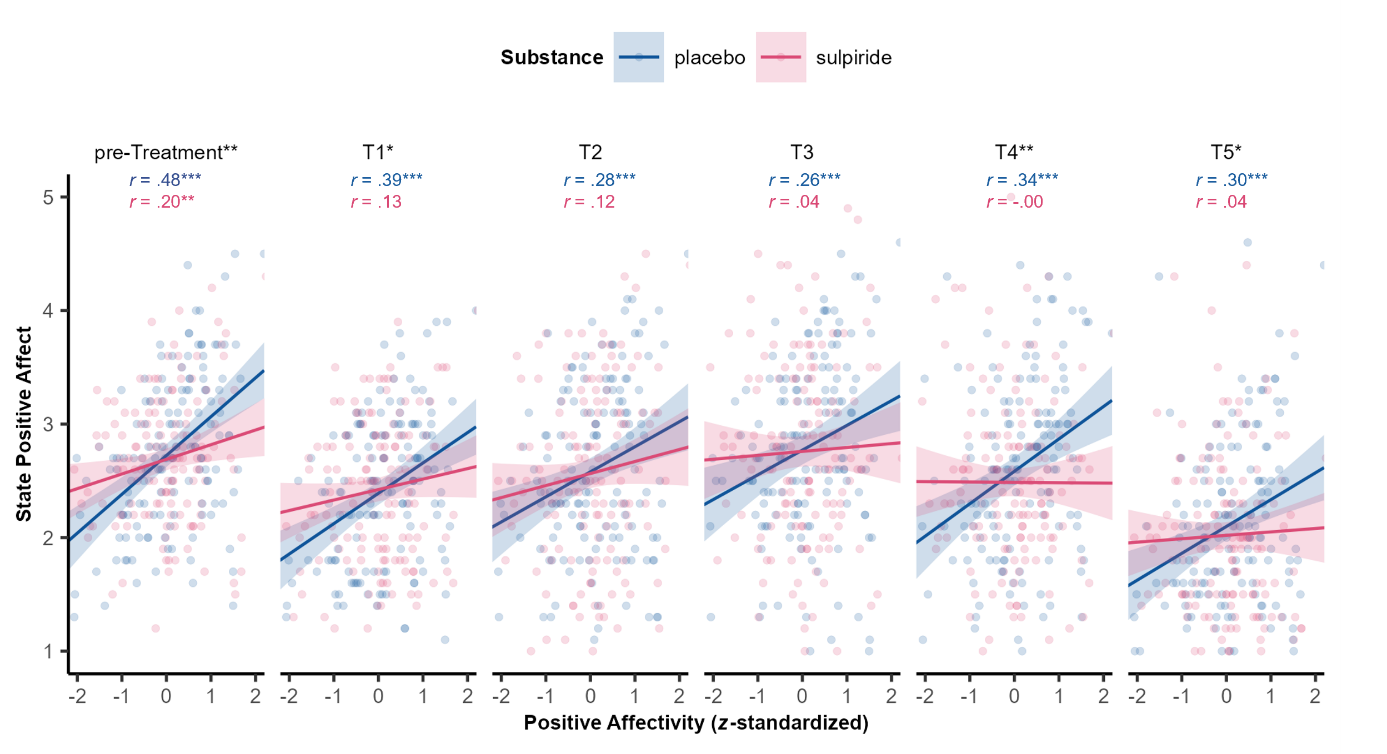
***

*Note.* State positive affect via PANAS contrasted with *z*-standardized Positive Affectivity and separated for substance groups. Black brackets indicate significantly different correlations. ^*^ *p < .05;* ^**^*p* < .01; ^***^ *p* < .001.

**Figure S3**

*State Positive Affect throughout the Experimental Session Predicted by Expectation Groups and Positive Affectivity*

**
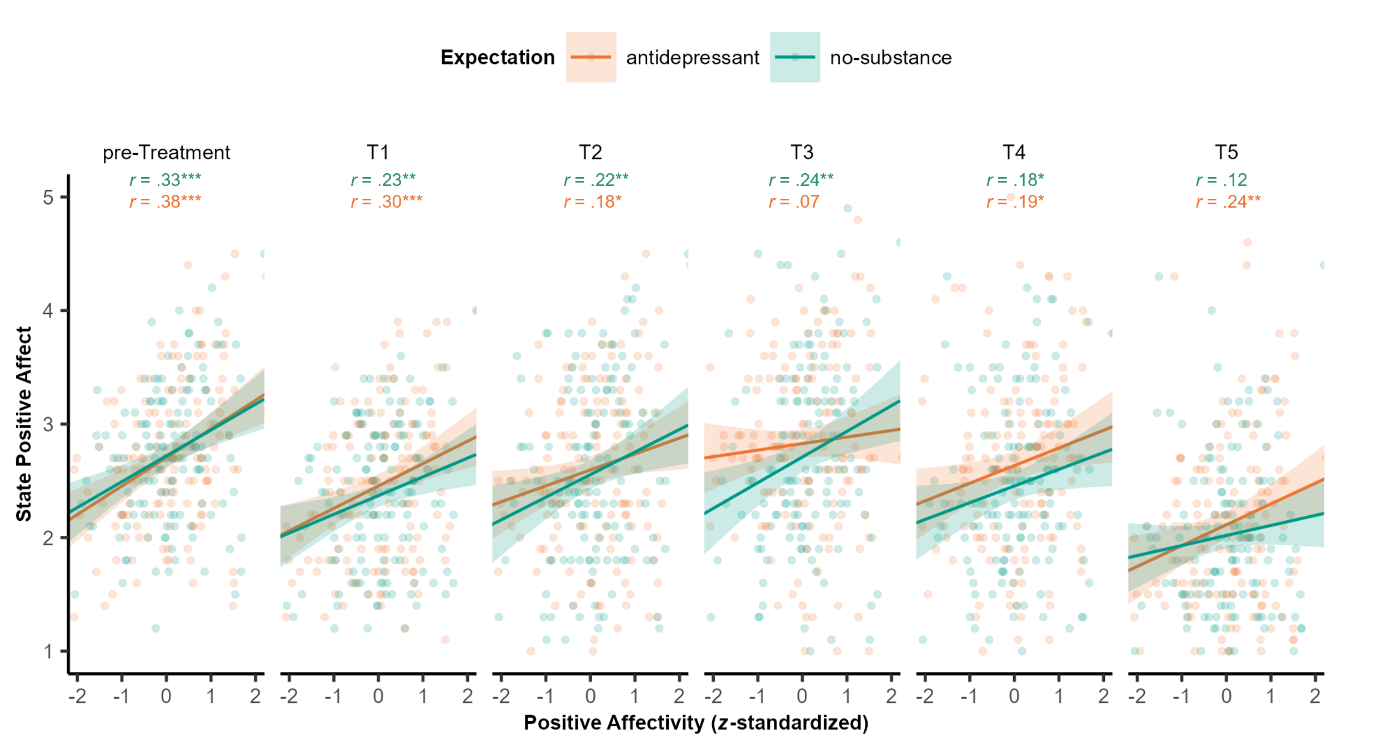
**

*Note.* State positive affect via PANAS contrasted with *z*-standardized Positive Affectivity and separated for expectation groups. ^*^ *p < .05;* ^**^*p* < .01; ^***^ *p* < .001

**Table S4**Correlation Coefficients of All Scales Included in the EFA

|  | MASQ_A | SHAPS | BFAS_E | BFAS_A | BDI-II | TEPS_C | TEPS_A | LOT-R_O | rBAS_RI | rBAS_GP | rBAS_RR | rBAS_I | BAS_D | BAS_FS | BAS_RR | PVSS |
| --- | --- | --- | --- | --- | --- | --- | --- | --- | --- | --- | --- | --- | --- | --- | --- | --- |
| MASQ_A | 1.00*** | 0.23** | -0.53*** | -0.38*** | 0.53*** | -0.23** | -0.33*** | -0.53*** | -0.52*** | -0.37*** | -0.48*** | -0.34*** | -0.31*** | -0.30*** | -0.28*** | -0.36*** |
| SHAPS | 0.23*** | 1.00*** | -0.26*** | -0.06 | 0.21* | -0.33*** | -0.30*** | -0.22** | -0.16 | -0.22** | -0.28*** | 0.02 | -0.14 | 0.05 | -0.13 | -0.35*** |
| BFAS_E | -0.53*** | -0.26*** | 1.00*** | 0.50*** | -0.38*** | 0.27*** | 0.29*** | 0.45*** | 0.55*** | 0.25** | 0.51*** | 0.37*** | 0.25** | 0.38*** | 0.24** | 0.36*** |
| BFAS_A | -0.38*** | -0.06 | 0.50*** | 1.00*** | -0.24** | 0.13 | 0.10 | 0.28*** | 0.50*** | 0.42*** | 0.33*** | 0.31*** | 0.37*** | 0.31*** | 0.23** | 0.21* |
| BDI-II | 0.53*** | 0.21*** | -0.38*** | -0.24*** | 1.00*** | -0.09 | -0.13 | -0.48*** | -0.37*** | -0.26*** | -0.25** | -0.07 | -0.22** | -0.16 | -0.15 | -0.26*** |
| TEPS_C | -0.23*** | -0.33*** | 0.27*** | 0.13* | -0.09 | 1.00*** | 0.45*** | 0.24** | 0.21** | 0.06 | 0.29*** | 0.17 | 0.08 | 0.12 | 0.17 | 0.26** |
| TEPS_A | -0.33*** | -0.30*** | 0.29*** | 0.10 | -0.13* | 0.45*** | 1.00*** | 0.20* | 0.26*** | 0.17 | 0.46*** | 0.20* | 0.24** | 0.21* | 0.40*** | 0.32*** |
| LOT-R_O | -0.53*** | -0.22*** | 0.45*** | 0.28*** | -0.48*** | 0.24*** | 0.20** | 1.00*** | 0.34*** | 0.33*** | 0.29*** | 0.12 | 0.33*** | 0.19* | 0.15 | 0.31*** |
| rBAS_RI | -0.52*** | -0.16** | 0.55*** | 0.50*** | -0.37*** | 0.21*** | 0.26*** | 0.34*** | 1.00*** | 0.42*** | 0.41*** | 0.47*** | 0.42*** | 0.56*** | 0.25** | 0.27*** |
| rBAS_GP | -0.37*** | -0.22*** | 0.25*** | 0.42*** | -0.26*** | 0.06 | 0.17** | 0.33*** | 0.42*** | 1.00*** | 0.36*** | 0.15 | 0.70*** | 0.07 | 0.27*** | 0.21** |
| rBAS_RR | -0.48*** | -0.28*** | 0.51*** | 0.33*** | -0.25*** | 0.29*** | 0.46*** | 0.29*** | 0.41*** | 0.36*** | 1.00*** | 0.42*** | 0.35*** | 0.30*** | 0.53*** | 0.39*** |
| rBAS_I | -0.34*** | 0.02 | 0.37*** | 0.31*** | -0.07 | 0.17** | 0.20*** | 0.12* | 0.47*** | 0.15** | 0.42*** | 1.00*** | 0.23** | 0.53*** | 0.24** | 0.19* |
| BAS_D | -0.31*** | -0.14* | 0.25*** | 0.37*** | -0.22*** | 0.08 | 0.24*** | 0.33*** | 0.42*** | 0.70*** | 0.35*** | 0.23*** | 1.00*** | 0.26*** | 0.36*** | 0.22** |
| BAS_FS | -0.30*** | 0.05 | 0.38*** | 0.31*** | -0.16** | 0.12* | 0.21*** | 0.19** | 0.56*** | 0.07 | 0.30*** | 0.53*** | 0.26*** | 1.00*** | 0.29*** | 0.15 |
| BAS_RR | -0.28*** | -0.13* | 0.24*** | 0.23*** | -0.15** | 0.17** | 0.40*** | 0.15* | 0.25*** | 0.27*** | 0.53*** | 0.24*** | 0.36*** | 0.29*** | 1.00*** | 0.30*** |
| PVSS | -0.36*** | -0.35*** | 0.36*** | 0.21*** | -0.26*** | 0.26*** | 0.32*** | 0.31*** | 0.27*** | 0.21*** | 0.39*** | 0.19** | 0.22*** | 0.15* | 0.30*** | 1.00*** |

*Note.* **p* < .05, ***p* < .01, ****p* < .001. MASQ_A = MASO-D30 Andedonic Depression; BFAS_E = BFAS-Enthusiasm; BFAS_A = BFAS-Assertiveness; TEPS_C = TEPS-Consummatory Pleasure; TEPS_A = TEPS-Anticipatory Pleasure; LOT-R_O = LOT-R Optimism; rBAS = RSTPQ-BAS; RI = Reward Interest; GP = Goal-Drive Persistence; RR = Reward Reactivity; I = Impulsivity; D = Drive; FS = Fun Seeking; RR = Reward Responsiveness.

***Specificity of Trait Anhedonia***

To test the specificity of anhedonia’s effect, separate tests were conducted with MASQ-D30 Anxious Arousal and General Distress subscales as continuous variables.

For General Distress, the omnibus test on the model yielded main effects of Time (*F*(5, 1419) = 82.92, *p* < .001, η^2^*_p_* = .226) and General Distress (*F*(1, 285) = 6.04, *p* = .015, η^2^*_p_* = .021). Like Anhedonia, General Distress was associated with reduced positive affect pre-treatment (*r*(286) = -0.15, *p* = .007). However, this association remained unchanged over time (*F*(5, 1419) = 0.88, *p* = .491, η^2^*_p_* = .003) and was not modulated by sulpiride (*F*(5, 1419) = 0.61, *p* = .691, η^2^*_p_* = .002). Rather, it persisted across substance groups over time (*r*(285) = -0.14, *p* = .016). No other effects emerged (all *p* > .096).

For Anxious Arousal, the test revealed a main effect of Time (*F*(5, 1419) = 82.78, *p* < .001, η^2^*_p_*  = .226) and a Substance × Anxious Arousal interaction, *F*(1, 285) = 6.70, *p* = .010, η^2^*_p_* = .023. Unlike Anhedonia, Anxious Arousal was not associated with positive affect pre-treatment (*r*(286) = -0.04, *p* = .520). Moreover, its interaction with Substance dissociated from the Substance × Anhedonia interaction: a negative association between anxious arousal and positive affect was observed over time for sulpiride (*r*(142) = -0.24, *p* = .004) but not placebo (*r*(141) = 0.09, *p* = .298). Thus, higher anxious arousal predicted reduced positive affect under sulpiride but not placebo. No further effects emerged (all *p* > .175).

***Anhedonia via SHAPS***

Similarly to the main results with the Andedonic Depression scores via the MASQ-D30 as the measure of trait anhedonia, the omnibus test with SHAPS-D yielded a main effect of Time (*F*(5, 1403) = 82.87, *p* < .001, η^2^*_p_* = .228), a main effect of Anhedonia (*F*(1, 281) = 6.62, *p* = .011, η^2^*_p_* = .023), and a Substance × Anhedonia interaction. Before treatment, there was a negative association between trait anhedonia and state positive affect across substance groups (*r*(285) = -0.14, *p* = .014). This negative association between trait anhedonia and state positive affect remained robust for the placebo substance group throughout the entire experimental session (*r*(141) = -0.25, *p* = .002), whereas it was not observed for the sulpiride substance group (*r*(142) = -0.04, *p* = .650). No other results reached significance (all *p* > .165).

***BID-II***

When the BDI-II was computed as the covariate, the omnibus test revealed a main effect of Time (*F*(5, 1419) = 81.53, *p* < .001, η^2^*_p_* = .223) and a main effect of BDI-II (*F*(1, 285) = 9.03, *p* = .003, η^2^*_p_* = .031). The internal consistency of the BDI-II in the present sample was satisfactory (Cronbach’s α = .84). No other effects were observed (all *p* > .192).

***TEPS***

To explore the consummatory and anticipatory facets of the TEPS, we computed separate tests with each subscale as covariate. For the consummatory facet, main effects of Time (*F*(5, 1419) = 83.99, *p* < .001, η^2^*_p_* = .228) and Consummatory Pleasure (*F*(1,285) = 4.13, *p* = .043, η^2^*_p_* = .014) were revealed. In addition, there were a trend Substance × Consummatory Pleasure interaction (*F*(1,285) = 3.80, *p* = .052, η^2^*_p_* = .013) and an Expectation × Substance × Time × Consummatory Pleasure interaction (*F*(5, 1419) = 2.28, *p* = .045, η^2^*_p_* = .008). No other effects emerged (all p > .052).

For Anticipatory Pleasure, a main effect of Time (*F*(5, 1419) = 81.12, *p* < .001, η^2^*_p_* = .222) and an Expectation × Substance × Time × Anticipatory Pleasure interaction (*F*(5, 1419) = 3.56, *p* = .003, η^2^*_p_* = .012) were found. No other effects emerged (all p > .070).

***Additional Items for Positive Affect***

We collected additional items from T1-T5 (i.e., after treatment and throughout the experimental session). Participants rated their affective states as defined by a list of adjectives (e.g., for positive affect: “happy, cheerful, amused, delighted”) on a 9-point Likert scale (0 = *not at all*, 8 = *very*). The rating battery also included unipolar scales for sadness (“depressed, saddened, sad, dejected”), fear (“afraid, fearful, filled with fear, scared”), and anger (“angry, furious, mad, incensed”), expectancy (“expectant”), as well as bipolar scales for unpleasant-pleasant, relaxed-nervous, tired-energetic, and bored-motivated.

For positive affect (“happy, cheerful, amused, delighted”), the omibus test revealed a main effect of Time (*F*(4, 1139) = 83.72, *p* < .001, η^2^*_p_* = .227), a main effect of Anhedonia (*F*(1, 285) = 28.38, *p* < .001, η^2^*_p_* = .091), a Substance × Time interaction (*F*(4, 1139) = 2.43, *p* = .046, η^2^*_p_* = .008), and a Substance × Anhedonia interaction (*F*(1, 285) = 6.22, *p* = .013, η^2^*_p_* = .021). No other results reached significance (all *p* > .144).

***Extraversion***

Although the Time × Substance × Extraversion interaction was not significant (*p* = .370), we wanted to explore whether the association between Extraversion and positive affect over time indicated a similar susceptibility to pharmacological manipulation as Anhedonia. To this end, Pearson correlations were computed for each time point and substance group. Before treatment, a positive correlation was observed for both placebo (*r*(141) = 0.36, *p* < .001) and sulpiride (*r*(143) = 0.18, *p* = .026). For placebo, this association was observed throughout (T1: *r*(143) = 0.27, *p* = .001; T3: *r*(143) = 0.17, *p* = .036; T4: *r*(143) = 0.26, *p* = .002; T5: *r*(143) = 0.21, *p* = .012), except at T2 (*r*(143) = 0.13, *p* = .107). For sulpiride, the correlation was no longer present post-treatment (T1: *r*(145) = 0.11, *p* = .178; T2: *r*(145) = 0.07, *p* = .402; T3: *r*(144) = -0.01, *p* = .951; T4: *r*(145) = -0.04, *p* = .592; T5: *r*(145) = 0.05, *p* = .526). Fisher’s *Z* tests indicated a significant difference between substance groups at T4, *Z* = 2.63, *p* = .009. No further effects emerged (all *p* > .149).

**State Negative Affect over Time**

***PANAS***

We tested if there were any effects on state negative affect as assessed via the negative affect scale of PANAS. Example items for included “distressed” and “upset”. Main effects of Time (*F*(5, 1419) = 50.05, *p* < .001, η^2^*_p_* = .150) and Anhedonia (*F*(1, 284) = 6.09, *p* = .014, η^2^*_p_* = .021) were revealed. Anhedonia was positively associated with state negative affect throughout the experimental session, *r*(285) = 0.14, *p* = .016. No other effects emerged (all *p* > .139).

***Additional Items for Sadness***

As described in *Additional Mood Items after Treatment*, we collected additional items from T1-T5. For sadness (i.e., “depressed, saddened, sad, dejected”), the omnibus test revealed a main effect of Time (*F*(4, 1139) = 164.21, *p* < .001, η^2^*_p_* = .366), a main effect of Anhedonia (*F*(1, 285) = 20.24, *p* < .001, η^2^*_p_* = .066), and a Anhedonia × Time interaction (*F*(4, 1139) = 3.71, *p* = .005, η^2^*_p_* = .013). In addition, there was a trend Substance × Anhedonia interaction (*F*(1, 285) = 3.64, *p* = .057, η^2^*_p_* = .013). No other effects emerged (all *p* > .152).

**Supplementary Reference**

1. Guilford, J.P. (1967). The nature of human intelligence. McGraw-Hill.
2. Basedow, L. A., Fischer, A., Benson, S., Bingel, U., Brassen, S., Büchel, C., ... & Rief, W. (2023). The influence of psychological traits and prior experience on treatment expectations. *Comprehensive Psychiatry*, *127*, 152431.
3. Rief, W., Barsky, A. J., Glombiewski, J. A., Nestoriuc, Y., Glaesmer, H., & Braehler, E. (2011). Assessing general side effects in clinical trials: Reference data from the general population. *Pharmacoepidemiology and Drug Safety*, 20(4), 405–415. https://doi.org/10.1002/pds.2067.
